# Association of Short-Term Exposure to Meteorological Factors and Risk of Hand, Foot, and Mouth Disease: A Systematic Review and Meta-Analysis

**DOI:** 10.3390/ijerph17218017

**Published:** 2020-10-30

**Authors:** Zhihui Liu, Yongna Meng, Hao Xiang, Yuanan Lu, Suyang Liu

**Affiliations:** 1 Department of Global Health, School of Health Sciences, Wuhan University, Wuhan 430071, China; zhizhiliu@whu.edu.cn (Z.L.); mengyongna@whu.edu.cn (Y.M.); xianghao@whu.edu.cn (H.X.); 2 Environmental Health Laboratory, Department of Public Health Sciences, University of Hawaii at Manoa, Honolulu, HI 96822, USA

**Keywords:** hand, foot, and mouth disease, infectious disease, meteorological factors, ambient temperature, relative humidity

## Abstract

(1) Background: Inconsistencies were observed in studies on the relationship between short-term exposure to meteorological factors and the risk of hand, foot, and mouth disease (HFMD). This systematic review and meta-analysis was aimed to assess the overall effects of meteorological factors on the incidence of HFMD to help clarify these inconsistencies and serve as a piece of evidence for policy makers to determine relevant risk factors. (2) Methods: Articles published as of 24 October 2020, were searched in the four databases, namely, PubMed, Web of Science, Embase, and MEDLINE. We applied a meta-analysis to assess the impact of ambient temperature, relative humidity, rainfall, wind speed, and sunshine duration on the incidence of HFMD. We conducted subgroup analyses by exposure metrics, exposure time resolution, regional climate, national income level, gender, and age as a way to seek the source of heterogeneity. (3) Results: Screening by the given inclusion and exclusion criteria, a total of 28 studies were included in the analysis. We observed that the incidence of HFMD based on the single-day lag model is significantly associated with ambient temperature, relative humidity, rainfall, and wind speed. In the cumulative lag model, ambient temperature and relative humidity significantly increased the incidence of HFMD as well. Subgroup analysis showed that extremely high temperature and relative humidity significantly increased the risk of HFMD. Temperate regions, high-income countries, and children under five years old are major risk factors for HFMD. (4) Conclusions: Our results suggest that various meteorological factors can increase the incidence of HFMD. Therefore, the general public, especially susceptible populations, should pay close attention to weather changes and take protective measures in advance.

## 1. Introduction

Hand, foot, and mouth disease (HFMD) is an airborne disease that causes a huge global disease burden [1]. HFMD was first discovered in New Zealand in 1957, and then gradually became known worldwide. In the past 20 years, HFMD outbreaks have become more and more common throughout the Asia-Pacific region [2,3]. Affected areas include, but are not limited to, Japan, South Korea, Singapore, Malaysia, Vietnam, and China. The main pathogens of HFMD are Coxsackie virus A16 (CA16) and Enterovirus 71 (EV71) [4]. Although HFMD is a self-limiting disease, its extensive harm to the human body cannot be ignored. The typical symptoms include fever, skin bursts on hands and feet, and vesicles in the mouth [2]. It is commonly seen in children between the ages of 0 and 15, especially children under five years of age [5]. Most HFMD patients suffer from complications such as myocarditis, pulmonary edema, aseptic meningoencephalitis, and even death. Therefore, identifying the environmental factors that are associated with HFMD has public health significance.

Over the past decade, the relationship between meteorological factors and HFMD has drawn widespread attention. However, the results are inconsistent, especially in terms of rainfall, wind speed, and sunshine duration. For example, Nguyen et al. (2017) [6] found that the increase in average weekly rainfall in nine provinces in the Mekong Delta region of Vietnam is associated with the increased risk of HFMD. However, Li et al. (2014) [7] observed no such association in Shanghai, China. Zhu et al. (2019) [8] found that the average daily sunshine duration is a protective factor for the risk of HFMD in Henan, China, while another similar Chinese study disapproved this observation in Shanghai, China [9]. Additionally, Xu et al. (2019) [10] found that the weekly minimum temperature was significantly associated with the increase the HFMD risk, while Hii et al. (2011) [11] found the weekly minimum temperature was a protective factor against HFMD. Yang et al. (2018) [12] and Hao et al. (2020) [13] observed that the daily average minimum relative humidity has an adverse effect on the risk of HFMD in Wuhan, China, while Nguyen et al. (2019) [14] found no such association in the Mekong Delta Region, Vietnam.

Due to inconsistent epidemiological studies mentioned above, we believe that a systematic review and meta-analysis may clarify these inconsistencies and help us see the big picture. Prior to this work, we were fully aware that a meta-analysis published in 2018 has done similar work [15]. However, we still decided to write this article for the following reasons: (1) After the publication of this study, a large number of epidemiological studies on HFMD and meteorological factors have been published in the scientific community, and the total number has doubled. (2) In the previous study, only two meteorological factors, ambient temperature and relative humidity, were included. In our study, rainfall, wind speed, and sunshine duration were also added into the analysis. (3) Our study not only includes effect estimates generated from single-day lag models, but also those from cumulative-day lag models. (4) In addition, we are interested in knowing whether the relationship between ambient temperature and relative humidity and HFMD risk has changed over time by comparing our results with ones reported by the previous meta-analysis.

With these concerns in mind, we conducted this study to systematically assess the impact of meteorological factors on the risk of HFMD, hoping to help policy makers identify high-risk factors and protect susceptible populations.

## 2. Methods 

### 2.1. Search Strategy

To find the related articles, we searched databases including PubMed, Web of Science, Embase, and MEDLINE using the following keywords: (“hand, foot and mouth disease” or “hand foot mouth disease” or “hand-foot-mouth disease” or “HFMD”) AND (“ambient temperature” or “temperature” or “humidity” or “relative humidity” or “climate” or “meteorology” or “weather” or “rainfall” or “precipitation” or “atmospheric pressure” or “barometric pressure “or “air pressure” or “wind speed” or “wind velocity” or “sunshine” or “sunshine duration”). Studies published up to the date of the search (24 October 2020) were considered. 

### 2.2. Eligibility and Selection Criteria

The inclusion criteria of our literature were as follows: (1) studies were written in English, or in other languages if an English translation was available; (2) studies provided effect estimates of relative risk (RR), incidence rate ratio (IRR), or the excess risk (ER); (3) studies reported cases of HFMD rather than death; (4) the associations were reported as effect estimates calculated based on one unit increase in meteorological factors (e.g., RR and 95% confidence intervals [Cls] of HFMD associated with each 1 °C increase in ambient temperature. Additionally, RRs that can be converted to standardized RR were also included [15,16]. 

The exclusion criteria of our literature were as follows: (1) the full text of the article could not be found; (2) the format of the paper is not a research article; (3) articles do not contain data for meta-analysis; and (4) articles do not estimate the effect of confounding from any source.

### 2.3. Data Extraction

The titles and abstracts derived from the databases have been downloaded to the reference manager Endnote (Version X8, developed by Clarivate Analytics, Philadelphia, United States) prior to the analysis. All records were independently evaluated by two investigators, and disputes are arbitrated by the third investigator. Upon removing the irrelevant articles and duplicate studies, the articles that met the selection criteria were included in our analysis. We extracted the following information from eligible articles: (1) study characteristics, (2) participants’ demographic factors, (3) the relative risks and 95% CLs of HFMD associated with meteorological factors, (4) outcomes of studies.

### 2.4. Quality Assessment

The quality of the selected articles was assessed by a Newcastle Ottawa Scale (NOS). The NOS included eight items in three dimensions [17]. Two investigators in our lab independently assessed the quality of the items according to the NOS guidelines, and the third arbitrator resolved the disagreement between two investigators if there was any. The results are shown in Supplementary Material Table S1. We only included articles with a NOS score of 6–9 to ensure that all studies included were of moderate or high quality.

### 2.5. The Selection of Lag Effects and Exposure Metrics

In selected studies, reporting associations on different lag days is a common practice. Among them, most studies were interested in single-day lag effects [6,11,18,19,20,21,22,23,24,25], while others focused on cumulative lag effects [12,26,27,28,29]. In our study, we selected lag days for analysis according to the criteria proposed by Atkinson et al. (2012) [30] and Yang et al. (2018) [31]. In short, if the results of an article were based on a single-day lag, then its results were included in the analysis completely. If an article was based on multiple lag days, we selected effects to be included in the analysis according to the following criteria: (1) the authors clearly indicated the priority of a certain lag, (2) the results were statistically significant on this lag, and (3) the lag days on which the associations of HFMD with both the maximum and minimum exposures were reported.

As for the exposure metrics, we selected results according to the following criteria. If only one exposure metric was presented (typically it is the mean exposure), then the effect of these metrics was included in the analysis. If associations of HFMD with the maximum and minimum exposure were reported rather than the mean exposure, then both were included. Additionally, if a study included results from more than one location, then results from all locations were included.

### 2.6. Statistical Analysis 

Forest plots were used to assess the heterogeneity of the associations of HFMD with temperature, relative humidity, wind speed, rainfall, and sunshine duration. Both the Cochran Q [32] and the I-squared (I^2^) statistics [33] were used to assess the heterogeneity. We conducted several subgroup analyses to explore the sources of heterogeneity, namely, gender (females vs. males), age (≤5 years vs. >5 years), exposure metrics (maximum vs. mean vs. minimum), exposed time resolution (daily vs. weekly vs. monthly), regional climate (tropical vs. temperate), and human development index (HDI) rank, a proxy for a country’s national income (high vs. low). The classification of HDI was based on the United Nations definition of low- and high-income countries [34]. To report the results, we mainly used RRs as the effect estimates. Two of the selected articles reported ER, and one reported IRR. We regarded them as RR, as they were approximately equal to RR [19].

Stata (version 12.0, Stata Corp. LLC, College Station, TX, USA) was used for data cleaning and merging. The “forest” package in R software (version 3.5.3, R Foundation for Statistical Computing, Vienna, Austria) was used to perform the meta-analysis. Publication bias was assessed by the Begg rank correlation test and Egger weighted regression test [35,36]. 

To assess the stability of the aggregated estimates, two sensitivity analyses were performed. The first sensitivity analysis was conducted to compare whether there is a difference in associations between meteorological factors and HFMD with extreme exposure values included or not, while the second sensitivity analysis was to compare the differences in effects, which has been previously reported by Cheng et al. (2018) [15] and effects generated by later updated literatures.

## 3. Results

### 3.1. Study Selection and Characteristics

**Figure 1:**
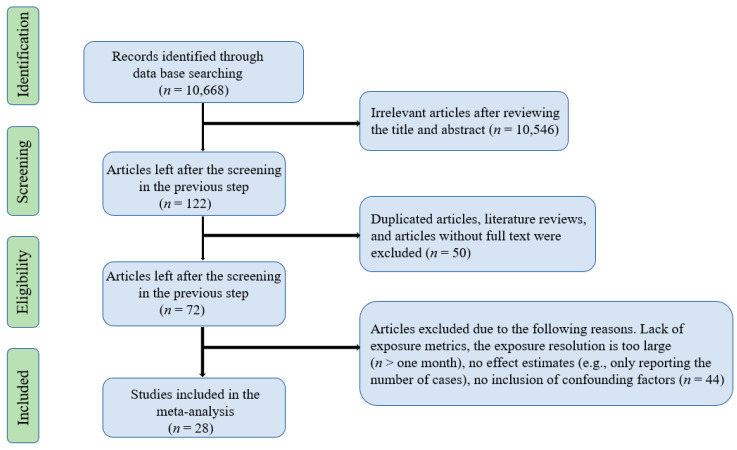
Flowchart showing the screening process for included articles.

A total of 10,668 records were searched through the first round of screening. After removing articles that did not meet the inclusion criteria, 28 studies were kept in this study. The screening process is presented in Figure 1. The characteristics of the selected articles were shown in Table 1. Among them, 25 studies assessed the associations of HFMD with ambient temperature, 23 with relative humidity, 11 with rainfall, 7 with wind speed, and 5 with sunshine duration for those focused on the single-day lag effect. Meanwhile, 14 studies assessed the associations of HFMD with ambient temperature and 10 studies with relative humidity based on cumulative lag models. The most commonly used statistical model is the generalized additive model (GAM). Case-crossover models and negative binomial regression are also used. Some articles used the distributed lag non-linear model (DLNM) and the above models together to assess the combined effect of time lags and meteorological factors on HFMD. All articles were conducted in Asia-Pacific regions. The majority of the selected articles were conducted in China, with the rest carried out in Japan, Korea, Singapore, and Vietnam.

### 3.2. The Overall Effects

**Figure 2:**
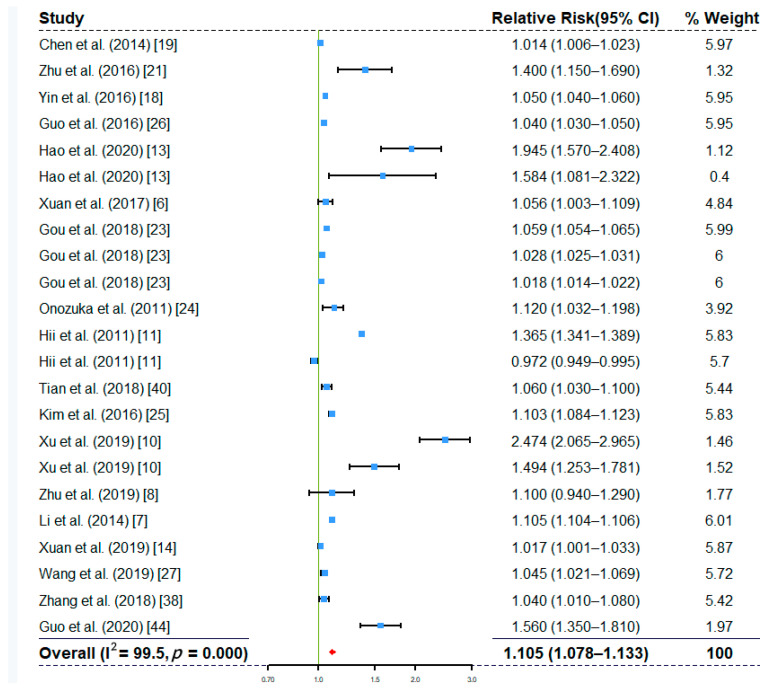
Forest plot showing the effect of ambient temperature on the risk of HFMD based on single-day lag models (*n* = 23).

**Figure 3:**
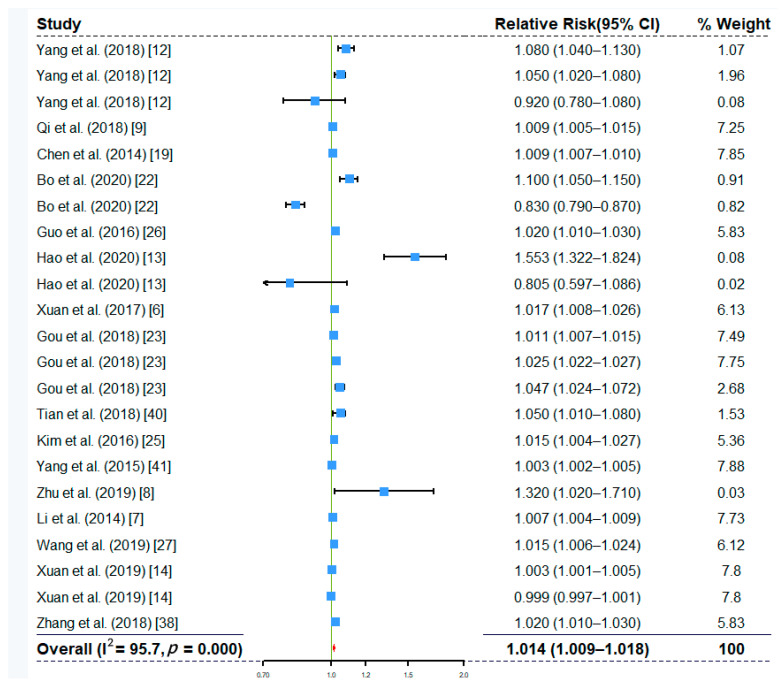
Forest plot showing the effect of relative humidity on the risk of HFMD based on single-day lag models (*n* = 23).

The overall effects of ambient temperature and relative humidity at single-day lags are shown in Figure 2 and Figure 3. The overall effects of other factors, including rainfall, wind speed, and sunshine duration, re shown in Figures S1–S3, respectively. The results suggested that ambient temperature (RR, 1.105; 95% CI, 1.078–1.133), relative humidity (RR, 1.014; 95% CI, 1.009–1.018), rainfall (RR, 1.001; 95% CI, 1.000–1.001), and wind speed (RR, 1.036; 95% CI, 1.013–1.059) had all been significantly associated with increased the risk of HFMD. The overall effect of sunshine duration (RR, 0.997; 95% CI, 0.975–1.018) on HFMD was not significant (Figure S3). The overall effects based on cumulative lag models also showed that ambient temperature (RR, 1.937; 95% CI, 1.564–2.400) and relative humidity (RR, 1.154; 95% CI, 1.030–1.294) were positively associated with the risk of HMFD (Figures S4 and S5). We observed not only significant associations of meteorological factors with HFMD risk, but also high heterogeneity (e.g., I^2^ is 99.6% for ambient temperature in Figure 2 and 95.7% for relative humidity in Figure 3). Therefore, we decided to conduct subgroup analyses to explore the sources of heterogeneity.

### 3.3. Subgroup Analyses

Our subgroup analysis focuses on the following variables to explore the sources of heterogeneity: exposure measures, exposure time resolution, regional climate, national income level, gender, and age. Table 2 and Table 3 presented results based on the single-day lag models, and Table S2 showed results based on cumulative-day lag models (due to lack of data, this table only contains results on ambient temperature and relative humidity).

#### 3.3.1. Measure of Meteorological Factors

In terms of ambient temperature, we found that for every 1 °C increase in average temperature, (*n* = 16), the incidence of HFMD increased by 5.7% (RR, 1.057; 95% CI, 1.030–1.084). Meanwhile, a 1 °C increase in maximum temperature (*n* = 4) was significantly associated with the incidence of HFMD (RR, 1.771; 95% CI, 1.355–2.315). There was no significant association between the minimum temperature (*n* = 3) and the incidence of HFMD. For relative humidity, a 1% increase in average (*n* = 13) and maximum (*n* = 5) relative humidity can both increase the incidence of HFMD (RR, 1.017; 95% CI, 1.011–1.024) and (RR, 1.015; 95% CI, 1.005–1.026), respectively. The minimum relative humidity (*n* = 4) had no significant association with the incidence of HFMD. As for rainfall and wind speed, the included articles mainly used average values, and they were all positively associated with the risk of HFMD. 

#### 3.3.2. Exposure Time Resolution

We observed that most of the included articles reported the RR values based on daily (*n* = 19) and weekly (*n* = 6) exposure values. Only one article used monthly exposure values to report results. Overall, for almost all meteorological factors, the RRs based on the daily and weekly exposure values are both significant (*p* < 0.05). Further, we found that the RRs generated by using the weekly exposure values are greater than the RRs produced by using the daily exposure values.

#### 3.3.3. Regional Climate

The included articles were based on two climate types, namely temperate climate and tropical climate. There were more studies carried out in the temperate zone than in the tropics. The results based on single-day lag models showed that ambient temperature and relative humidity were significantly associated with HFMD risk in both temperate and tropical zones. A further observation revealed that the effects of ambient temperature, relative humidity, and rainfall on the risk of HFMD in the temperate zone are higher than that in the tropics. Meanwhile, the RR produced in the temperate zone under the cumulative lag model is also much higher than that in the tropical zone (Table S2).

#### 3.3.4. Human Development Index (HDI)

Since there was no relevant data on national income, we used HDI as a proxy. Most of the included studies were conducted in areas with high HDI. We found that in the high HDI areas, ambient temperature, relative humidity, and rainfall were significantly associated with the risk of HFMD. Among them, the effect of ambient temperature and relative humidity on HFMD was higher in the high HDI areas than in the low HDI areas. The effect of rainfall in the high HDI areas was lower than those in the low HDI regions, but this effect was not statistically significant.

#### 3.3.5. Gender and Age

Regarding ambient temperature and relative humidity, more than one third of the included articles reported the risks by gender and age groups. The effects of ambient temperature and relative humidity on HFMD were statistically significant in both genders, and there were no observable differences in the effect size of the two. For age groups, children under five are at higher risk than those over five.

### 3.4. Sensitivity Analysis and Publication Bias

We conducted two sensitivity analyses. In the first sensitivity analysis, we removed the extreme exposure values (maximum and minimum values) to verify whether the overall effect was affected by the extreme values. The results showed that the effects with extremes removed are comparable to those with extremes retained in the analysis, except for the ambient temperature, which had a higher effect on the risk of HFMD with extremes included than without them included (Table S3). Since a previous article has done a meta-analysis regarding meteorological factors and HFMD risk [15], our second sensitivity analysis aimed to compare the differences in the results reported in this article and results reflected in the updated literature. Comparing the two, we found that the overall effect of ambient temperature on HFMD risk has strengthened, while no obvious trend was observed for relative humidity over time (Table S4). We used both Begg’s and Egger’s tests to check whether publication bias exists. The results showed that articles regarding ambient temperature and relative humidity had some degrees of publication bias. The bias was not found on other meteorological factors (Table S5).

## 4. Discussion

This systematic review and meta-analysis explored the relationship between meteorological factors and the incidence of HFMD. We found that ambient temperature, relative humidity, rainfall, and wind speed were all significantly associated with the risk of HFMD. Further subgroup analyses also revealed that temperate regions, high-income countries, and children under five years of age are at high risk of developing HFMD. This article is the first study to include rainfall, wind speed, and sunshine duration into a meta-analysis of meteorological factors and HFMD, and it is also the first article to take the cumulative effect into the analysis.

We observed that both the maximum and mean ambient temperatures were significantly associated with the incidence of HFMD, and the effect of the maximum temperature was stronger than that of the mean temperature, which is consistent with the results of the previous meta-analysis [15]. Our sensitivity analysis also confirmed this result; that is, after removing the extreme high temperature from the analysis, the effect size is reduced (Table S3). Laboratory studies have found that high temperature can increase the infectivity of enteroviruses [45], and the virus multiplies faster in the high temperature environment as compared to that in the low temperature environment [46,47,48]. In hot weather, the public may increase outdoor activities, especially for children who are more likely to be infected through contact with recreational facilities and contaminated food [49,50]. Meanwhile, our results are consistent with most previous studies that the maximum and the mean relative humidity are positively associated with the incidence of HFMD. Moderate humidity may help the virus adhere to the surface of small objects, such as toys and food, which facilitates the survival and spread of the virus [51]. In addition, higher humidity affects the metabolism of children, limiting their sweat excretion and reducing their immunity [12].

Consistent with the result of [15], we found that the size of effect estimates generated by the use of weekly data are larger than the ones generated by the use of daily data. This may be due to the incubation period of the disease. HFMD has a short incubation period (typically three to seven days) and a long viral infection period. It can exist in infected people and people who have just recovered, which causes the second episode [52]. Therefore, there will always be a delay between when a patient notices the symptoms and this patient visits the doctor [53]. The delayed response to a clinical visit will affect the spread of the disease within a certain period of time [54]. Therefore, when measuring risk through the use of weekly data, it is easier to capture the association due to the time lags caused by the incubation period. For smaller geographic areas with low population density (such as rural areas), the use of weekly data can better investigate this association and improve statistical power [15].

Conventionally, HFMD is regarded as a tropical disease, though we found a higher meteorologically associated risk in temperate areas than in the tropics. This may be due to the various weather conditions in different climatic zones [26]. In temperate countries, the outbreak of HFMD usually occurs in summer or early fall [11]. Compared with tropical regions, temperate climates are more suitable for the survival and reproduction of enteroviruses. People living in temperate regions may be more involved in outdoor activities, thereby increasing their exposure to the virus. In contrast, although the commonly occurring rainy season in the tropics helps improve the breeding of the virus [55], once the rainfall exceeds a threshold, it will instead reduce people’s outdoor activities, thereby reducing the spread of the virus [11]. This may explain that the highest incidence of HFMD was not in the tropics, but temperate areas. In addition, the frequency of extreme weather events occurring in the temperate zone was much lower than that in the tropics, which provides a good environment for the survival and reproduction of the virus.

Our study found that in high-income countries, ambient temperature, relative humidity, and rainfall all had a significant effect on the incidence of HFMD, while the previous meta-analysis did not find such statistical significance [15]. The possible reason is that the classification method of income level is different. Since there is no suitable standard, we used the 2019 United Nations Human Development Index as the basis to divide countries into high- and low-income countries. Our classification method has resulted in more areas defined as high-income countries, which increases the statistical power to detect the associations. Meanwhile, the high risk observed in high-income countries may also be due to people having better medical resources and stronger testing capabilities [18,56,57,58]. In high-income countries, almost all cases are recorded into the system, with few missed cases. This helps find the true associations with less selection bias. In the subsequent sensitivity analysis, we tried to classify income levels similar to what the previous meta-analysis had applied and divided countries into high- and middle-income countries and observed comparable results for the high-income countries (Table S4).

For the subgroup analysis on gender and age, we found that the risk of HFMD did not differ between genders. Children under five are at higher risk than those older than five. This may be due to their imperfect immune system development [59,60]. It has been reported that children under five years old lack antibodies such as Cox A16 and EV 71 to HFMD [61,62]. In addition, children sharing toys between playmates can also promote the spread of the virus [18,63].

We conducted two sensitivity analyses. The first sensitivity analysis showed that the overall effect of including extreme temperatures is higher than that of excluding extreme temperatures (Table S3), which showed that it is important to prevent HFMD when extreme temperature events occur. The second sensitivity analysis showed that the association between meteorological factors and the risk of HFMD increased over time (Table S4). One possible reason is that global warming in recent years has led to higher ambient temperatures, which in turn has accelerated the spread of enteroviruses, thus increasing the risk of HFMD [64].

Our paper has the following limitations. First, we observed significant publication bias, especially on ambient temperature and relative humidity. The main reason for publication bias is that researchers tend to report statistically significant results and ignore those that are not significant. Some approaches to solve the problem of publication bias include: (1) trim and fill, (2) selection models, and (3) meta-regression. However, all of them have their own flaws. For example, the trim and fill method requires the elimination of articles that affect the symmetry of the funnel plot. The second method uses a selection model to screen the included articles, assuming that the probability of publication is associated with the *p* value of its results, which ignores other factors that may affect publication. Meta-regression requires a large number of samples for the calculation to make sense [65]. Therefore, we chose to leave the publication bias as is in order to maintain the authenticity of the results. Relevant to the first limitation, our second limitation is that our results are highly heterogeneous, despite the fact that we used subgroup analysis to explore the source and sensitivity analysis to reduce the heterogeneity. Most of the included articles were carried out based on second-hand data, and they tended not to report insignificant results, even if such results did exist. As a result, we were not able to include these insignificant effects in our analysis. Third, we noticed that most of the included studies were conducted in the Asia-Pacific region, and there is currently a lack of relevant studies from other regions of the world. This may be another form of selection bias. Based on the above limitations, cautions need to be taken when our results are generalized to other regions of the world.

## 5. Conclusions

This systematic review and meta-analysis found that ambient temperature, relative humidity, rainfall, and wind speed were statistically significantly associated with HFMD. Extremely high temperature and relative humidity significantly increased the risk of HFMD. The overall impact of ambient temperature on the risk of HFMD has increased over time. Policy makers need to take preventive measures to deal with the impact of climate factors on the incidence of HFMD. Future research should focus on the non-Asia-Pacific region and relatively less studied meteorological factors, such as rainfall, wind speed, and sunshine duration.

## Figures and Tables

**Table 1:** Characteristics of the included studies on the associations between meteorological factors and the risk of HFMD.

Reference	Study Location	Study Period	Population	Ages	Exposure Variable	Statistical Model	Temporal Lags	Resolution	Climate Group	Measure Index	HDI Rank	Quality Scores	Outcome
Zhu et al. (2015) [29]	Shandong, China	2007–2012	108,377	0–5 years	Cumulative maximum temperature; cumulative minimum temperature	Distributed lag non-linear model (DLNM) with Poisson distribution, adjusting for relative humidity, rainfall, sunshine duration, DOW, public holidays, seasonal trend, and long trend	0–14 days	Daily	Temperate climate	RR	High	8	Reported HFMD
Chen et al. (2014) [19]	Guangzhou, China	2009–2011	34,527	0–14 years	Mean temperature; relative humidity	Generalized additive model (GAM), adjusting for long-term trend, seasonal trend, day of week, and public holidays	0–10 days	Daily	Temperate climate	IRR	High	7	Reported HFMD
Yang et al. (2018) [12]	Hefei, China	2011–2016	NA	All	Mean temperature; rainfall; cumulative mean relative humidity	DLNM, adjusting for long-term trend, seasonal trend, and day of week	0–20 days	Daily	Temperate climate	RR	High	8	Reported HFMD
Xu et al. (2015) [37]	Beijing, China	2010–2012	113,475	6–15 years	Mean temperature; relative humidity; cumulative maximum temperature; cumulative minimum temperature	A newly developed case-crossover design with DLNM, adjusting for day of week, public holidays, long-term trend, and seasonal trend	0–13 days	Daily	Temperate climate	RRs	High	7	Reported HFMD
Yu et al. (2018) [28]	Guilin, China	2014–2016	88,742	0–14 years	Relative humidity; sunshine duration; wind speed; rainfall; cumulative maximum temperature; cumulative minimum temperature; cumulative maximum relative humidity; cumulative minimum relative humidity;	DLNM, adjusting for long-term trends, seasonality, differences in the annual at-risk population; day of week, and public holidays	0–14 days	Daily	Temperate climate	RR	High	7	Reported HFMD
Zhang et al. (2018) [38]	Henan, China	2012–2013	NA	0–5 years	Mean temperature; relative humidity; rainfall; sunshine duration; wind speed	Bayesian space–time hierarchy mode, Poisson with log link regression and GeoDetector, adjusting for long-term trend and autocorrelation	None	None	Temperate climate	RR	High	6	Reported HFMD
Qi et al. (2018) [9]	Shanghai, China	2009–2015	51,776	0–15 years	Mean temperature; relative humidity	DLNM, adjusting for potential confounders of long time trend, DOW, and public holidays	0–14 days	Daily	Temperate climate	RR	High	8	Reported HFMD
Zhu et al. (2016) [21]	Shandong, China	2007–2012	504,017	0–5 years	Mean temperature	DLNM, adjusting for seasonal trend, long time trend, DOW, and public holidays	0–21 days	Daily	Temperate climate	RR	High	9	Reported HFMD
Bo et al. (2020) [22]	143city, China	2009–2014	3,060,450	0–12 years	Relative humidity	DLNM, adjusting for long-term trends, seasonality, autocorrelation, DOW public holidays	0–18 days	Daily	None	RR	High	9	Reported HFMD
Wang et al. (2016) [39]	Hong Kong, China	2009–2014	1534	All	Rainfall; wind speed; sunshine duration; cumulative mean temperature: cumulative maximum relative cumulative minimum relative humidity	A combination of negative binomial generalized additive models and DLNM, adjusting for multiple environmental factors, long-term trends, and seasonality	0–30 days	Daily	Tropical climate	RR	High	7	Reported HFMD
Yin et al. (2016) [18]	Chengdu, China	2010–2013	76,403	0–14 years	Mean temperature	DLNM, adjusting for seasonal trend, long time trend, DOW, and holidays	0–13 days	Daily	Temperate climate	RR	High	7	Reported HFMD
Guo et al. (2016) [26]	Guangdong, China	2009–2013	400,408	0–14 years	Relative humidity; cumulative mean temperature; cumulative mean relative humidity	A mixed generalized additive models (MGAM), adjusting for seasonal trend, long time trend, DOW, and holidays	0–14 days	Daily	Temperate climate	RR	High	7	Reported HFMD
Hao et al. (2020) [13]	Wuhan, China	2013–2017	NA	All	Mean temperature; cumulative maximum temperature; cumulative minimum temperature; relative humidity; cumulative maximum relative humidity; cumulative minimum relative humidity	DLNM combined with Poisson regression, adjusting for DOW, seasonality, and long-term time trend	0–14 days	Daily	Temperate climate	RR	High	7	Reported HFMD
Xuan et al. (2017) [6]	Can Tho, Vietnam	2012–2014	NA	All	Mean temperature; relative humidity	Time-series regression analysis, adjusting for seasonality, long-term time trend, DOW, and the offset of population	0–6 days	Daily	Tropical climate	ER	Low	7	Reported HFMD
Gou et al. (2018) [23]	Gansu, China	2010–2014	NA	All	Mean temperature; relative humidity	Generalized linear regression models (GLM) with Poisson link and classification and regression trees (CART), adjusting for seasonality	0–12 weeks	Weekly	Temperate climate	ER	High	6	Reported HFMD
Onozuka et al. (2011) [24]	Fukuoka, Japan	2000–2010	73,684	All	Mean temperature; relative humidity	Negative binomial regression, adjusting for seasonal and inter-annual variations	0–3 weeks	Weekly	Temperate climate	RR	High	7	Reported HFMD
Hii et al. (2011) [11]	Singapore	2001–2008	NA	All	Maximum temperature minimum temperature	Time series Poisson regression models, adjusting for seasonality, long-term time trend, and autocorrelation	0–2 weeks	Weekly	Temperate climate	RR	High	8	Reported HFMD
Tian et al. (2018) [40]	Beijing, China	2010–2012	114,777	0–4 years	Mean temperature; relative humidity; wind speed; sunshine duration	Bayesian spatiotemporal Poisson regression models; adjusting for seasonality and inter-annual variations	None	None	Temperate climate	RR	High	7	Reported HFMD
Kim et al. (2016) [25]	South Korea	2010–2013	214,642	All	Mean temperature; relative humidity	GAM and Poisson distribution, controlling for seasonality, long-term time trend, and autocorrelation	0–2 weeks	Weekly	Temperate climate	RR	High	8	Reported HFMD
Xuan et al. (2019) [14]	Mekong Delta region, Vietnam	2014–2016	NA	0–5 years	Mean temperature; humidity; cumulative rainfall	DLNM with quasi-Poisson, controlling for long-term trend and autocorrelation	0–20 days	Daily	Temperate climate	RR	High	7	Reported HFMD
Li et al. (2014) [7]	Guangzhou, China	2009–2012	166,770	All	Mean temperature; relative humidity	Negative binomial multivariable regression, adjusting for long-term trend and autocorrelation	None	Weekly	Temperate climate	ER	High	6	Reported HFMD
Xu et al. (2019) [10]	Guangdong, China	2010–2013	1,048,574	0–5 years	Mean temperature; maximum temperature; minimum temperature; mean relative humidity; mean wind speed; rainfall; sunshine duration; cumulative maximum temperature; cumulative minimum temperature; cumulative mean temperature	DLNM with quasi-Poisson, controlling for long-term trend and autocorrelation	0–21 days	Daily	Temperate climate	RR	High	7	Reported HFMD
Yang et al. (2015) [41]	Hefei, China	2010-2012	21,634	0–14 years	Relative humidity	Poisson linear regression model and DLNM, adjusting for mean temperature, seasonal patterns, and long-term trends, day of week	0–21 days	Daily	Temperate climate	ER	High	7	Reported HFMD
Zhu et al. (2019) [8]	Xiamen, China	2013–2017	36,464	All	Mean temperature; relative humidity; sunshine duration	DLNM with quasi-Poisson, adjusting for long-term time trend, DOW, and public holidays	0–20 days	Daily	Temperate climate	RR	High	7	Reported HFMD
Wang et al. (2019) [27]	Guangdong, China	2009–2012	911,640	All	Mean temperature; mean relative humidity; mean rainfall	Bayesian spatiotemporal model autocorrelation, adjusting for long-term time trend and autocorrelation	None	Monthly	Temperate climate	RR	High	7	Reported HFMD
Zhu et al. (2020) [42]	Wuxi, China	2011–2017	107,906	All	Cumulative maximum temperature; cumulative minimum temperature	DLNM, adjusting for time-varying factors and other meteorological factors	0–16 days	Daily	Temperate climate	RR	High	7	Reported HFMD
Ji et al. (2020) [43]	Tianjin, China	2014–2018	70,027	0–15 years	Cumulative mean temperature	DLNM and a susceptible infectious recovery models, adjusting for long-term trends, seasonality, DOW	0–14 days	Daily	Temperate climate	RR	High	8	Reported HFMD
Guo et al. (2020) [44]	Xi’an, China	2009–2018	31,2018	All	Maximum temperature; cumulative maximum temperature	DLNM, combined with the GAM, adjusting for long-term trends and seasonality, and week	0–8 weeks	Weekly	Temperate climate	RR	High	6	Reported HFMD

**Table 2:** Subgroup analysis for the increased HFMD risk associated with ambient temperature and relative humidity based on single-day lag models.

Subgroup Types	Ambient Temperature			Relative Humidity		
	*n*	I^2^%, *p*-Value	Pooled RR (95% CI)	*n*	I^2^%, *p*-Value	Pooled RR (95% CI)
Measure						
Mean	15	99.6%, *p* = 0.000	1.057 (1.030–1.084)	13	95.8%, *p* = 0.000	1.017 (1.011–1.024)
Maximum	4	94.5%, *p* = 0.000	1.771 (1.355–2.315)	5	94.7%, *p* = 0.000	1.015 (1.005–1.026)
Minimum	3	93.0%, *p* = 0.000	1.288 (0.896–1.853)	4	95.0%, *p* = 0.000	0.899 (0.782–1.034)
Time resolution						
Daily	8	92.8%, *p* = 0.000	1.074 (1.038–1.111)	15	93.7%, *p* = 0.000	1.009 (1.004–1.014)
Weekly	12	99.7%, *p* = 0.000	1.121 (1.084–1.161)	5	96.4%, *p* = 0.000	1.018 (1.008–1.028)
Monthly	1		1.045 (1.021–1.069)	1		1.015 (1.006–1.024)
Regional climate						
Tropical	4	99.6%, *p* = 0.000	1.093 (0.917–1.303)	3	89.8%, *p* = 0.000	1.004 (0.999–1.009)
Temperate	15	99.6%, *p* = 0.000	1.103 (1.074–1.133)	18	95.1%, *p* = 0.000	1.017 (1.012–1.022)
HDI *						
High	21	99.6%, *p* = 0.000	1.114 (1.085–1.144)	20	95.5%, *p* = 0.000	1.016 (1.011–1.022)
Low	2	49.1%, *p* = 0.161	1.028 (0.994–1.063)	3	89.8%, *p* = 0.000	1.004 (0.999–1.009)
Gender						
Male	5	97.2%, *p* = 0.000	1.195 (1.085–1.317)	5	86.3%, *p* = 0.007	1.008 (1.002–1.014)
Female	5	96.8%, *p* = 0.000	1.196 (1.073–1.334)	5	84.3%, *p* = 0.007	1.006 (1.000–1.012)
Age						
0–5 year	10	95.1%. *p* = 0.000	1.101 (1.052–1.152)	10	83.9%, *p* = 0.000	1.010 (1.004–1.016)
>5 year	8	77.9%, *p* = 0.000	1.037 (0.996–1.080)	6	16.7%, *p* = 0.306	1.002 (0.999–1.006)

* HDI: Human Development Index.

**Table 3:** Subgroup analysis for the increased HFMD risk associated with rainfall, wind speed, and sunshine duration based on single-day lag models.

Subgroup Types	Rainfall			Wind Speed			Sunshine Duration		
	*n*	I^2^%, *p*-Value	Pooled RR (95%CI)	*n*	I^2^%, *p*-Value	Pooled RR (95%CI)	*n*	I^2^%, *p*-Value	Pooled RR (95% CI)
Measure									
Mean	10	97.6%, *p* = 0.000	1.001 (1.000–1.001)	5	95.6%, *p* = 0.000	1.075 (1.023–1.129)			
Maximum	1		1.001 (1.000–1.002)	1		1.040 (1.030–1.050)			
Minimum				1		1.010 (1.006–1.014)			
Time resolution									
Daily	4	84.3%, *p* = 0.000	0.999 (0.998–1.001)	2	88.1%, *p* = 0.004	1.001 (0.792–1.265)	2	95.0%, *p* = 0.000	0.984 (0.951–1.018)
Weekly	6	98.6%, *p* = 0.000	1.001 (1.001–1.002)	1		1.016 (1.006–1.026)	1		1.200 (1.054–1.366)
Monthly	1		1.004 (1.001–1.008)	1		0.990 (0.982–0.998)	1		0.997 (0.985–1.009)
Regional climate									
Tropical	2	76.5%, *p* = 0.039	1.003 (0.999–1.007)						
Temperate	10	97.6%, *p* = 0.000	1.001 (1.000–1.001)						
HDI *									
High	9	97.8%, *p* = 0.000	1.001 (1.000–1.001)						
Low	2	76.5%, *p* = 0.039	1.003 (0.999–1.007)						

* HDI: Human Development Index.

## Supplementary Materials

The following are available online at https://www.mdpi.com/1660-4601/17/21/8017/s1: Table S1: The quality of articles assessed by Newcastle Ottawa Scale (NOS), Table S2: Subgroup analysis for the increased HFMD risk associated with ambient temperature and relative humidity based on cumulative lag models, Table S3: The overall effect of each meteorological factor on the risk of HFMD (with or without extreme exposure values), Table S4: The differences in the effects of meteorological factors on HFMD between two meta-analyses (Chen et al. (2018) V.S the current study), Table S5: The *p* values of Begg’s and Egger’s tests by meteorological factors based on single- and cumulative-day lag models, Tabl: Forest plot showing the effect of rainfall on the risk of HFMD based on single-day lag models (*n* = 11), Figure S2: Forest plot showing the effect of wind speed on the risk of HFMD based on single-day lag models (*n* = 7), Figure S3: Forest plot showing the effect of sunshine duration on the risk of HFMD based on single-day lag models (*n* = 5), Figure S4: Forest plot showing the effect of ambient temperature on the risk of HFMD based on cumulative-day lag models (*n* = 18), Figure S5: Forest plot showing the effect of relative humidity on the risk of HFMD based on cumulative-day lag models (*n* = 10).
