# Supplementary material for: Association of Short-Term Exposure to Meteorological Factors and Risk of Hand, Foot, and Mouth Disease: A Systematic Review and Meta-Analysis
Source: Int J Environ Res Public Health. 2020 Oct 30;17(21):8017. doi: 10.3390/ijerph17218017 (PMC7663009; doi:10.3390/ijerph17218017)
Supplement: Supplementary file 1 [file ijerph-17-08017-s001.pdf]

Table S1. The quality of articles assessed by Newcastle Ottawa Scale (NOS).

First author. (Year)	Quality assessment (NOS)			Total score
	Selection	Comparability	Outcome	
Zhu et al. (2015)	3	2	3	8
Chen et al. (2014)	3	2	2	7
Yang et al. (2018)	3	2	3	8
Xu et al. (2015)	3	2	2	7
Yu et al. (2018)	3	2	2	7
Zhang et al. (2018)	3	1	2	6
Qi et al. (2018)	3	2	3	8
Zhu et al. (2016)	4	2	3	9
Bo et al. (2020)	4	2	3	9
Wang et al. (2016)	3	2	2	7
Yin et al. (2016)	3	2	2	7
Guo et al. (2016)	3	2	2	7
Hao et al. (2020)	3	2	2	7
Xuan et al. (2017)	3	2	2	7
Gou et al. (2018)	3	1	2	6
Onozuka et al. (2011)	3	1	3	7
Hii et al. (2011)	4	1	3	8
Tian et al. (2018)	3	2	2	7
Kim et al. (2016)	4	2	2	8
Wang et al. (2019)	4	1	2	7
Xuan et al. (2019)	4	2	2	8
Xu et al. (2019)	4	1	2	7
Yang et al. (2015)	3	2	2	7
Zhu et al. (2019)	3	2	2	7
Li et al. (2014)	3	1	2	6
Zhu et al. (2020)	3	1	3	7
Ji et al. (2020)	3	2	3	8
Gou et al. (2020)	3	1	2	6

Table S2. Subgroup analysis for the increased HFMD risk associated with ambient temperature and relative humidity based on cumulative lag models.

Subgroup types	Ambient temperature			Relative humidity		
	n	I^2%, p-value	Pooled RR (95% CI)	n	I^2%, p-value	Pooled RR (95%CI)
**Measure**						
Mean	5	90.3%, p = 0.000	1.473 (1.147-1.892)	4	54.7%, p = 0.085	1.154 (1.053-1.264)
Maximum	8	95.5%, p = 0.000	2.660 (2.028-3.488)	3	90.2%, p = 0.000	1.406 (0.996-1.985)
Minimum	5	84.6%, p = 0.000	1.443 (1.092-1.906)	3	91.1%, p = 0.000	1.042 (0.740-1.468)
**Regional climate**						
Tropical	1		1.420 (0.540-3.732)	1		2.762 (1.636-4.662)
Temperate	17	97.2%, p = 0.000	1.955 (1.573-2.429)	7	89.1%, p = 0.000	1.110 (0.996-1.237)

Table S3. The overall effect of each meteorological factor on the risk of HFMD (with or without extreme exposure values).

Variable	N[1]	Pooled RR (95% CI)[1]	N[2]	Pooled RR (95% CI)[2]
**Single-day lag**				
ambient temperature	23	1.105 (1.078- 1.133)	15	1.060 (1.033-1.088)
relative humidity	23	1.014 (1.009-1.018)	13	1.016 (1.012-1.021)
rainfall	12	1.001 (1.000-1.001)	10	1.001 (1.000-1.001)
wind speed	7	1.036 (1.013-1.059)	5	1.075 (1.023-1.129)
sunshine duration	5	0.997 (0.975-1.018)	5	0.997 (0.975-1.018)
**Cumulative-day lag**				
ambient temperature	18	1.937 (1.564-2.400)	5	1.468 (1.206-1.787)
relative humidity	10	1.154 (1.030-1.294)	4	1.154 (1.053-1.264)

[1]: With extreme exposure values included; [2]: With extreme exposure values excluded.

Table S4. The differences in the effects of meteorological factors on HFMD between two meta-analyses (Chen et al. (2018) V.S the current study).

Subgroup types	Ambient Temperature				Relative humidity			
	N[1]	Pooled RR (95% CI)	N[2]	Pooled RR (95% CI)	N[1]	Pooled RR (95% CI)	N[2]	Pooled RR (95% CI)
**Measure**								
Mean	7	1.05 (1.03–1.08)*	15	1.060 (1.033-1.088)*			13	1.017 (1.011-1.024)*
Maximum	1	1.18 (0.82–1.53)*	3	1.859 (1.235-2.798)*			5	1.015 (1.005-1.026)*
Minimum	1	0.90 (0.76–1.03)*	3	1.288 (0.896-1.853)			4	0.899 (0.782-1.034)*
**Time resolution**								
Daily	5	1.02 (1.01–1.03)*	7	1.048 (1.018-1.079)*	5	1.01 (1.00–1.01)*	15	1.009 (1.004-1.014)*
Weekly	5	1.07 (1.00–1.13)*	12	1.121 (1.084-1.161)*	3	1.02 (0.99–1.06)	5	1.018 (1.008-1.028)*
Monthly	1	1.07 (1.05–1.09)*	1	1.045 (1.021-1.069)*	1	1.03 (1.02–1.04)*	1	1.015 (1.006-1.024)*
**Regional climate**								
Tropical	3	1.02 (0.89–1.16)	1	1.017 (1.001-1.033)*	1	1.03 (1.02–1.04)*	3	1.004 (0.999-1.009)
Subtropical	4	1.06 (1.02–1.10)*	7	1.137 (1.089-1.188)*	5	1.01 (1.00–1.01)*	15	1.014 (1.009-1.018)*
Temperate	1	1.02 (1.01–1.03)*	4	1.048 (1.029-1.067)*	1	1.00 (1.00–1.01)*	3	1.021 (1.009-1.034)*
**National income**								
High	4	1.04 (0.93–1.15)	4	1.132 (0.963-1.330)*	2	1.05 (1.02–1.07)*	2	1.029 (0.999-1.601)*
Middle	5	1.05 (1.02–1.08)*	17	1.065 (1.047-1.083)*	5	1.01 (1.01–1.01)*	18	1.015 (1.009-1.021)*

[1]: Articles included by Chen et al. (2018); [2]: Later updated articles that were not included in the study of Chen et al. (2018).

Table S5. The P values of Begg's and Egger's tests by meteorological factors based on single- and cumulative-day lag models.

Variable	Begg's Test	Egger's test
**Single-day lags**		
Ambient temperature	0.001	0.331-
Relative humidity	0.032	0.013
Rainfall	0.945	0.38
Wind speed	0.23	0.082
Sunshine duration	0.806	0.904
**Cumulative-day lags**		
Temperature	0.405	0.343
Relative humidity	0.152	0.269

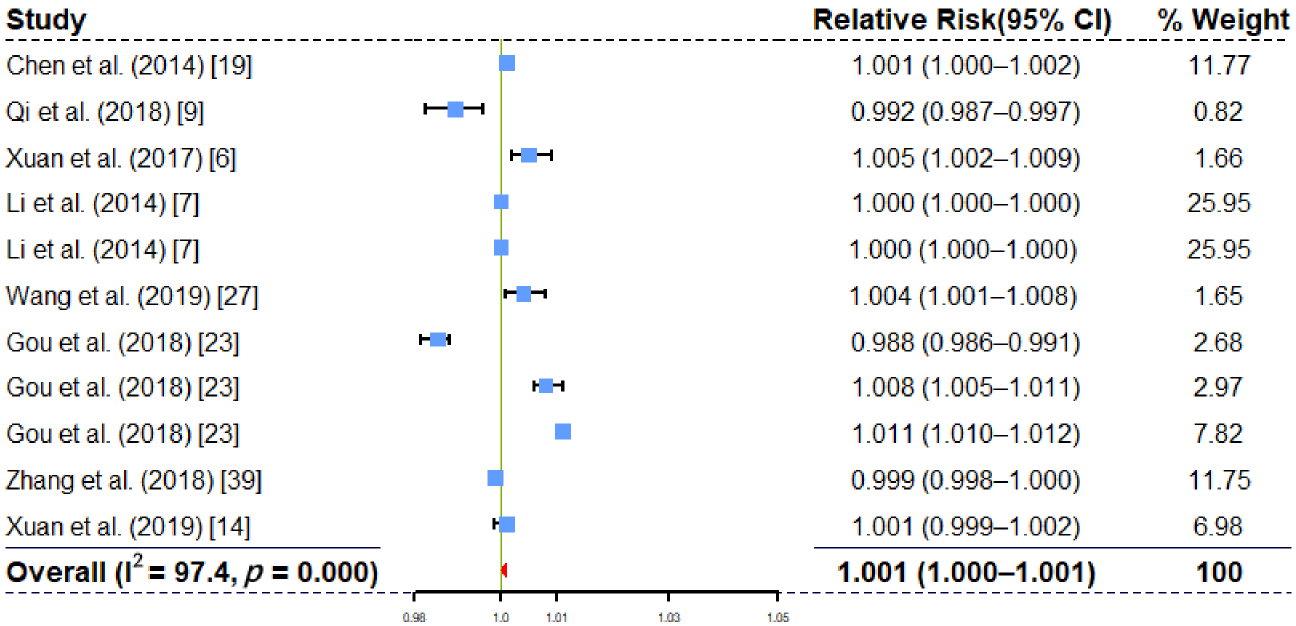

Figure S1. Forest plot showing the effect of rainfall on the risk of HFMD based on single-day lag models (n = 11).

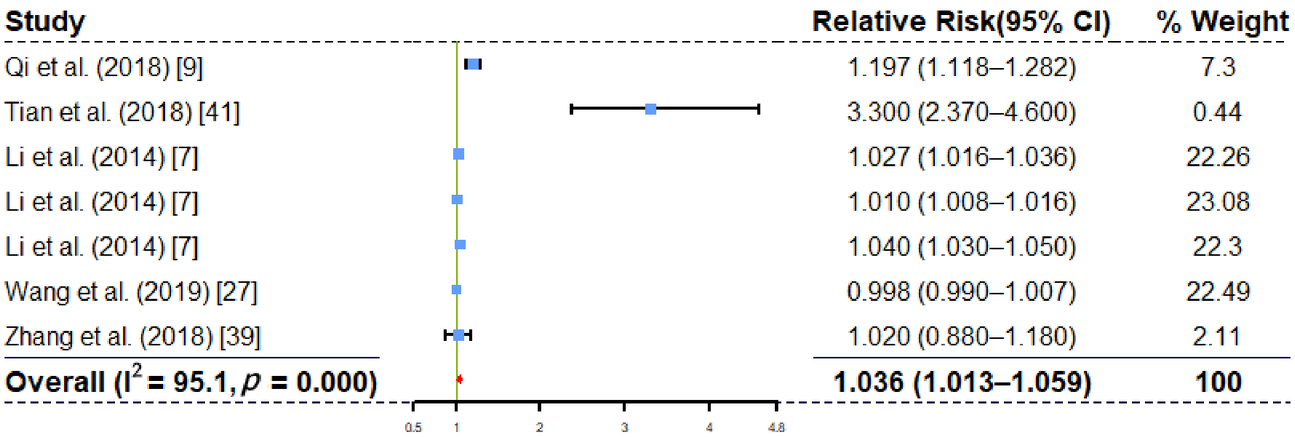

Figure S2. Forest plot showing the effect of wind speed on the risk of HFMD based on single-day lag models (n = 7).

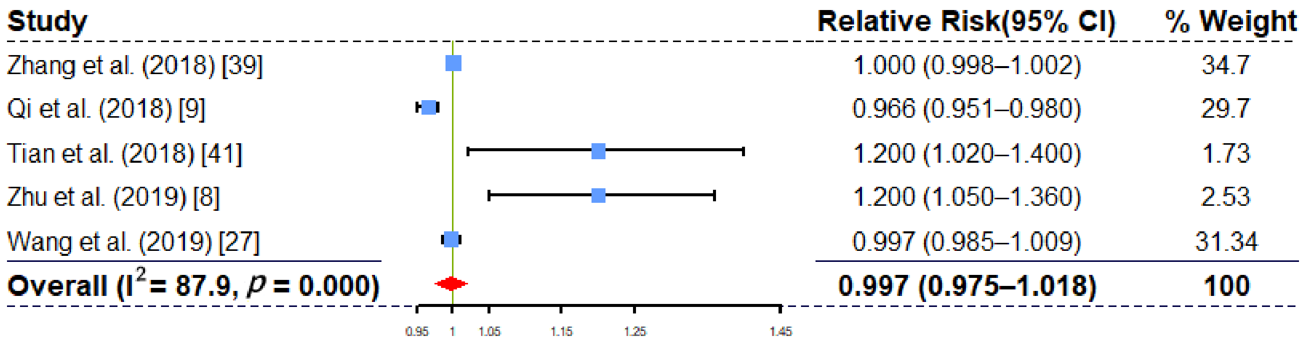

Figure S3. Forest plot showing the effect of sunshine duration on the risk of HFMD based on single-day lag models (n = 5).

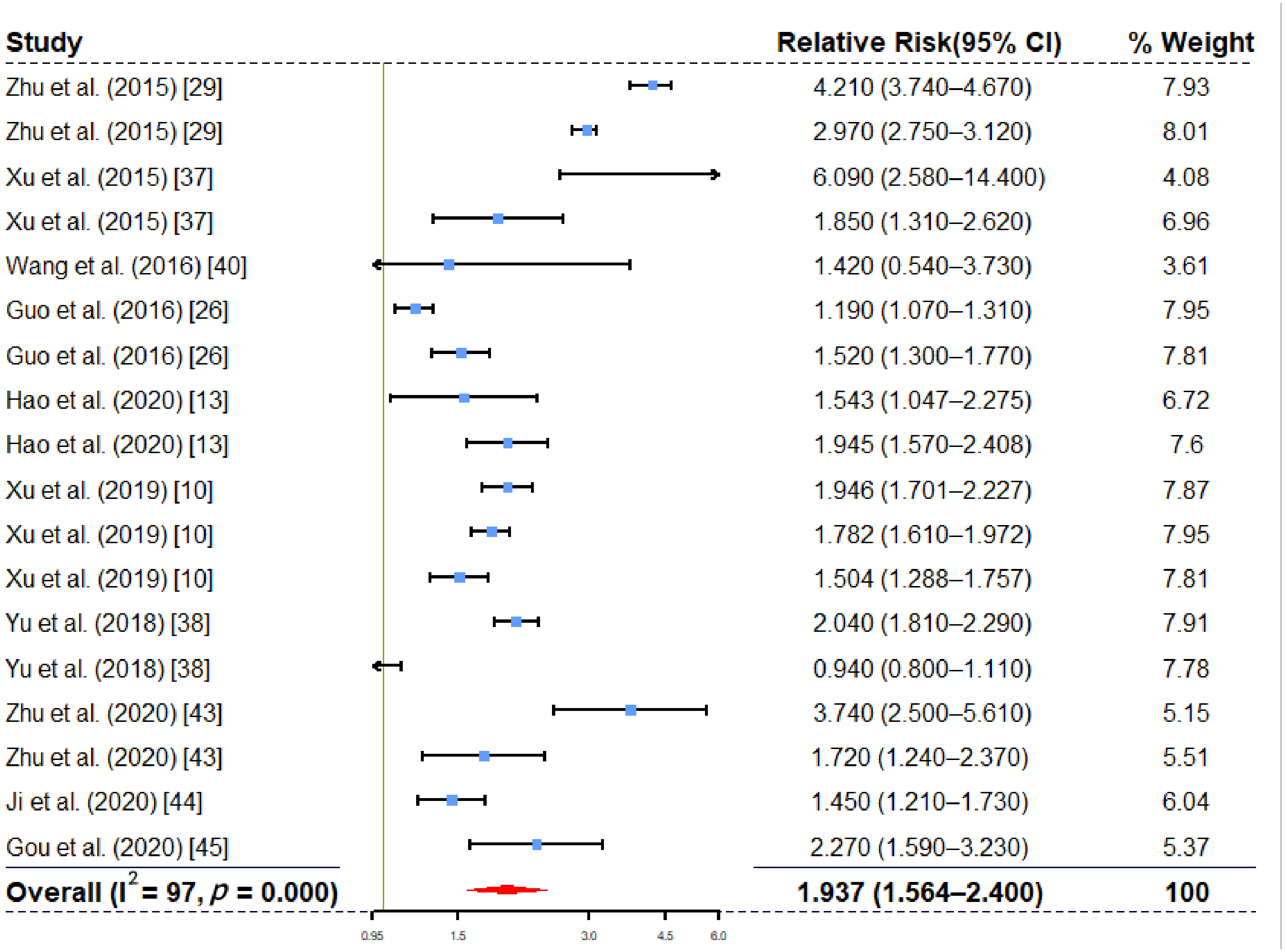

Figure S4. Forest plot showing the effect of ambient temperature on the risk of HFMD based on cumulative-day lag models (n = 18).

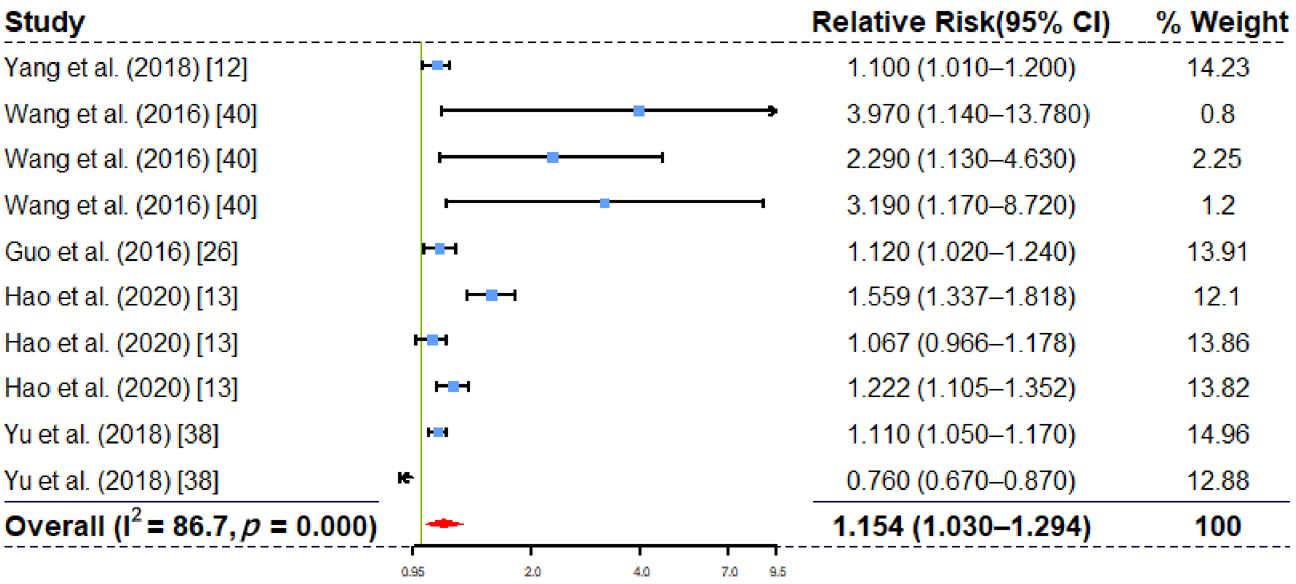

Figure S5. Forest plot showing the effect of relative humidity on the risk of HFMD based on cumulative-day lag models (n = 10).
